# Inter-sectoral approaches for the prevention and control of malaria among the mobile and migrant populations: a scoping review

**DOI:** 10.1186/s12936-018-2562-4

**Published:** 2018-11-16

**Authors:** Cho Naing, Maxine A. Whittaker, Marcel Tanner

**Affiliations:** 1 Institute for Research, Development and Innovation (IRDI), International Medical University, Kuala Lumpur, Malaysia; 2 Division of Tropical Health and Medicine, James Cook University, Townsville, QLD Australia; 3 Swiss Tropical and Public Health Institute, Basel, Switzerland

**Keywords:** Malaria, Mobile, Migrants, Intersectoral, Interventions, Review

## Abstract

**Background:**

Malaria cases among mobile and migrant populations (MMPs) represent a large and important reservoir for transmission, if undetected or untreated. The objectives of this review were to identify which intersectoral actions have been taken and how they are applied to interventions targeted at the MMPs and also to assess the effect of interventions targeted to these special groups of population.

**Results:**

A total of 36 studies met the inclusion criteria for this review. Numerous stakeholders were identified as involved in the intersectoral actions to defeat malaria amongst MMPs. Almost all studies discussed the involvement of Ministry of Health/Public Health (MOH/MOPH). The most frequently assessed intervention among the studies that were included was the coverage and utilization of insecticide-treated nets as personal protective measures (40.5%), followed by the intervention of early diagnoses and treatment of malaria (33.3%), the surveillance and response activities (13.9%) and the behaviour change communication (8.3%). There is a dearth of information on how these stakeholders shared roles and responsibilities for implementation, and about the channels of communication between-and-within the partners and with the MOH/MOPH. Despite limited details in the studies, the intermediate outcomes showed some evidence that the intersectoral collaborations contributed to improvement in knowledge about malaria, initiation and promotion of bed nets utilization, increased access to diagnosis and treatment in a surveillance context and contributed towards a reduction in malaria transmission. Overall, a high proportion of the targeted MMPs was equipped with correct knowledge about malaria transmission (70%, 95% CI 57–83%). Interventions targeting the use of bed nets utilization were two times more likely to reduce malaria incidence amongst the targeted MMPs (summary OR 2.01, 95% CI 1.43–2.6) than the non-users. The various intersectoral actions were often more vertically organized and not fully integrated in a systemic way within a given country or sub-national administrative setting.

**Conclusion:**

Findings suggest that interventions supported by the multiple stakeholders had a significant impact on the reduction of malaria transmission amongst the targeted MMPs. Well-designed studies from different countries are recommended to robustly assess the role of intersectoral interventions targeted to MMPs and their impact on the reduction of transmission.

**Electronic supplementary material:**

The online version of this article (10.1186/s12936-018-2562-4) contains supplementary material, which is available to authorized users.

## Background

The ultimate goal of the Global Technical Malaria Strategy 2016–2030 is to eliminate malaria from at least 35 countries by 2030 [1, 2]. In 2016, 91 countries reported on the indigenous malaria cases. Among these, 15 countries carried 80% of the global malaria burden [3]. In some of the pre-elimination countries, malaria is now limited to remote, forested areas, and often malaria cases are largely found in mobile and migrant populations (MMPs) [4]. The link between malaria transmission and human population movement (HPM) has been acknowledged many years ago [5]. Historically, it has been noted that the failure to consider HPM has been one factor contributing to the failure of malaria eradication campaigns in the 1950s and the 1960s [6–9].

As transmission declines due to concerted efforts of malaria control, it often becomes increasingly focal [10] or found as pockets of transmission [11]. Control programmes should target the remaining parasite reservoirs, deploying resources with increasing granularity [10] to populations who are at high risk of malaria transmission. This often includes MMPs. Numerous studies have reported that MMPs face many obstacles in accessing equitable essential healthcare services due to their living and working conditions, education level, gender, illegal migration status, language and cultural barriers, anti-migrant sentiments and lack of migrant-inclusive health policies, among others [12–14]. In the context of achieving and sustaining malaria elimination, there is a need to have health services that are used by MMPs and this requires specific service-delivery because they move into and through multiple localities that may have different malaria transmission levels and risks [13].

Efforts should be directed towards implementation of integrated interventions through multilateral partnerships across health and non-health sectors [12]. However, there are limited and mixed evidences about the success of intersectoral malaria-focussed activities and HPM. For instance, some studies reported there was no clear linkages between the health sectors and other sectoral ministries [14], while other studies showed reduction of malaria incidence through intersectoral activities [15]. Additionally, descriptions of successful intersectoral approaches to malaria in general, and in particular for MMPs are limited. Intersectoral interventions (activities/actions) for malaria in this review refers to the inclusion of several sectors in addition to the health sector when designing and implementing public policies to improve quality of life [16] for MMPs.

The current study address the research question: What sectors are addressing and implementing intervention(s) targeted towards malaria control of the MMPs?

The objectives were to:

- Identify what intersectoral actions have been taken and how they are applied to intervention(s) targeted at the MMPs,
- Establish which intervention(s) targeted to these special group of populations is/are effective and
- Identify the knowledge gaps and lessons learned about the interventions focused upon MMPs.

This systematic review was commissioned by the WHO/TDR (2017/721367-0).

## Methods

**Fig. 1:**
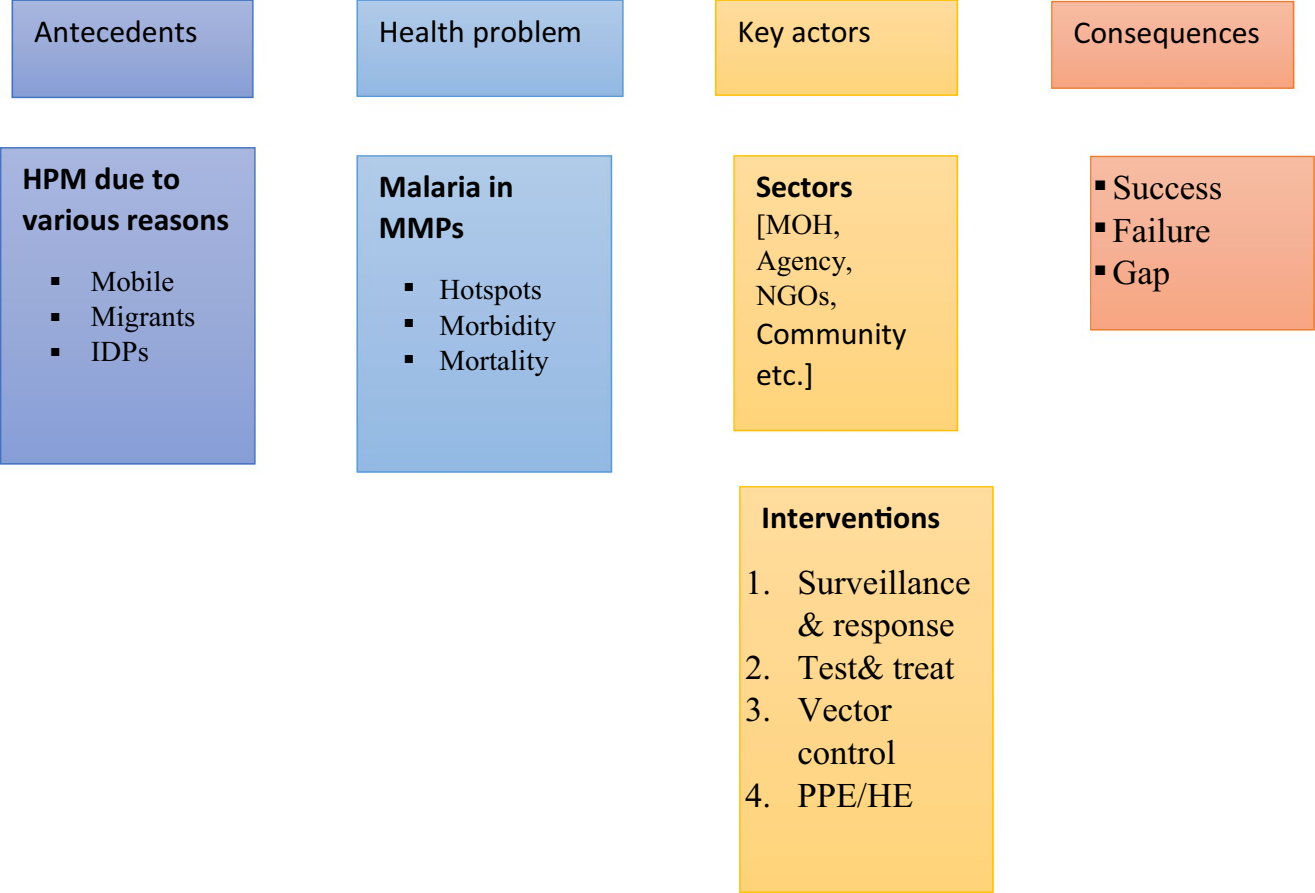
A conceptual framework for intersectoral activities addressing malaria and human population movement

The current review was carried out, following the Preferred Reporting Items for Systematic Reviews and Meta-Analyses (PRISMA) guideline [17] (Additional file 1). A conceptual framework for intersectoral activities addressing malaria and HPM is provided (Fig. 1). The framework identified three main domains that can contribute to the consequences of malaria interventions targeted towards the HPM. The domains included are the antecedents, the health problems and the key actors. The first domain, the antecedents which were considered in the present review include mobile population, migrants and IDPs. In general, these vulnerable populations have encountered multiple health problems. However, the focus of this study is primarily on malaria. The key factors that are involved in the implementation of interventions are also described. The two inter-linked factors are then identified as the sectors and the interventions involved where multiple sectors are involved. The sectors involved are broadly categorized as MOH, other ministries/non-health sectors (e.g., labour/social welfare department, immigrations departments, agriculture departments), agencies, non-governmental organizations (NGOs), community and so forth. The interventions where these sectors are involved are classified as (i) surveillance and response, (ii) test and treat, (iii) vector control and (iv) PPE/HE, in accordance with the global malaria control strategy. All these three domains are sequentially linked which contribute to the consequences of interventions implemented by the sectors, which are targeted to the HPM. The consequences are broadly identified as success (achieved the programme target), failure (unmet targets) or gap between the expectations and actual achievements of the sectors involved.

### Study search

The relevant studies were searched in the health-related databases, such as PubMed, Medline, Embase, ProQuest, Global Health, and Google Scholar. Keywords used in the search included: malaria, *Plasmodium falciparum*, *Plasmodium vivax,* migrants, migration, hard-to-reach, marginalised, multisectoral, intersectoral, stakeholders with the use of Boolean operator, truncating and proximity operators, as appropriate. The search was extended to the WHO websites for Roll Back Malaria Partnership to end malaria (RBM) documents, the international agency websites including International Organization for Migration (IOM), United Nations High Commission for Refugees (UNHCR), United Nations Children’s Fund (UNICEF) and the international NGOs websites including Population Services International (PSI).

### Study selection

The selection criteria for the current review are provided as population, concepts and context in the PCC format [18].

#### Population (P)

**S**tudies with participants having malaria and categorized as MMP, were included regardless of age, gender and their legal status. As theories and definitions of migration are diverse [11] and migration is not a definitive risk for malaria [19], the MMP in this review is defined in the context of malaria, rather than general definition of MMP. In the present study MMPs are defined as “*individuals who move to and/or from the endemic/studied areas for a certain period of time and live and/or work at a certain distance from forest and/or forest*-*like settings”* [20]. This can include internally displaced persons (IDPs) (4), defined as individuals who have been forced to leave their homes or places of habitual residence, in particular, as a result of or in order to avoid the effects of armed conflict, situations of generalized violence, violations of human rights, or natural or man-made disasters, and who have not crossed an international border [21]. The present study classified the collective movement of all MMPs including IDPs as human population movement (HPM) [22].

#### Concepts (C)

All interventions targeting to the prevention or control of malaria were included. The interventions were summarized into five categories; (i) surveillance and response to surveillance, (ii) test and treat, (iii) health education/promotion, (iv) personal prevention and (v) vector control.

#### Contents (C)

Published and unpublished epidemiologic studies were considered, assessing interventions for malaria control, case studies (publications that describe implementation of interventions), position papers (publications that focus on policy) as well as a relevant narrative review (publication that include the description of actual or proposed interventions) with a focus on intersectoral collaboration for malaria control among MMPs. The following outcomes of intersectoral interventions targeted at MMPs were considered.

Interventions that

- Benefited participants (levels of knowledge, attitudes and practices of malaria control),
- Demonstrated positive behaviour changes with significant reduction in malaria incidence,
- Had increased detection of asymptomatic malaria cases,
- Demonstrated intersectoral coordination (qualitatively or quantitatively).

The search was limited to publications in English language between 1978 and 2017, regardless of the study location. An initial search was performed in February 2017, and repeated in July 2017 and May 2018 to update the study search. Articles that were primarily concerned with other issues rather than intersectoral collaboration to address malaria amongst MMPs were excluded.

### Data extraction

Several steps were involved in data extraction in the present review. First, two investigators individually screened the titles and abstracts, and then selected full-text articles, according to the selection criteria. The two investigators independently extracted information from each included study using a data extraction form prepared for the review. The data extraction form had been pre-tested by the investigators on a sample of papers to check its utility, comprehensiveness and ease of use. Any discrepancy was resolved by consensus. Information collected were: first author and publication year, methods (design, year of data collection), location (country of study, setting), participants (sample size, characteristics), intersectoral action (sectors involved), interventions, outcomes, mechanisms for intersectoral action. For studies with qualitative information, the two investigators independently reviewed each article for a second time and then, coded for the major/prominent themes such as lessons learned for ‘success’ and/or ‘challenges’ encountered.

### Data synthesis

Details of the included studies were combined as a narrative review by the domain of outcomes. If there were a minimum of three studies reporting the outcomes in similar ways, a meta-analysis of outcome data was performed. For qualitative information, the results from each theme were summarized in a tabular format. No judgment was made on the methodological quality of the included studies, as many of these were cross-sectional descriptive surveys, surveillance reports or retrospective chart/record reviews. Instead, the analyses were stratified by interventions identified.

## Results

**Fig. 2:**
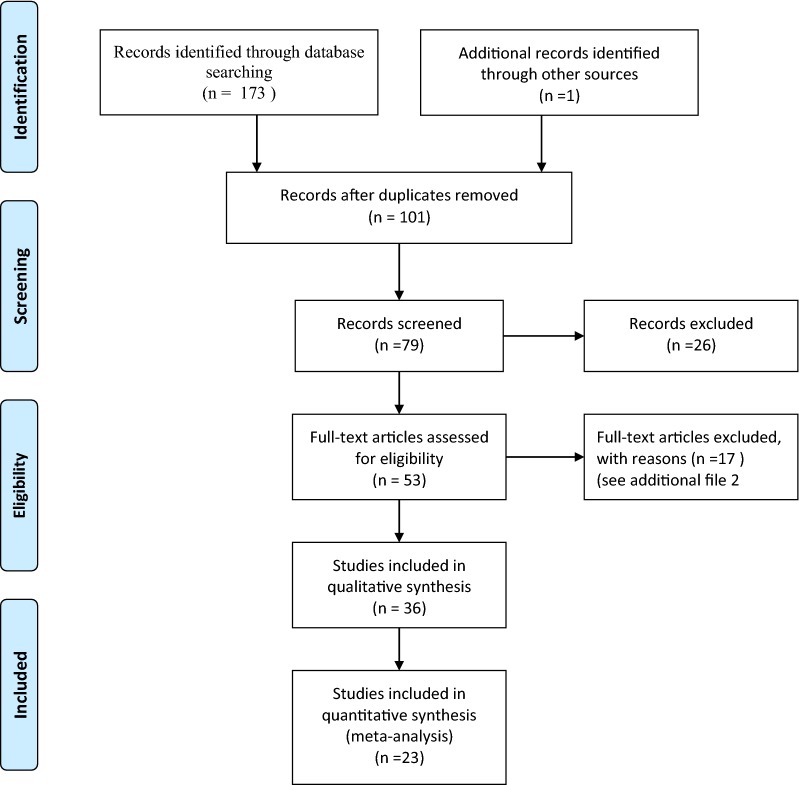
Study selection flowchart

Figure 2 shows the four-phase PRISMA flow chart of the study selection process. The initial search yielded 174 citations. After the title and abstract screening, a total of 53 studies were considered and a final of 36 studies met our inclusion criteria [15, 20, 22–55]. A list of seventeen excluded studies along with the main reasons for exclusion is provided in Additional file 2. Table 1 provides the characteristics of the included studies. Of these studies, the vast majority of studies were cross-sectional descriptive surveys (78%, 28/36), four were case studies, two were reviews, and one study each was randomized trial and evaluation report.

**Table 1:** Characteristics of the included studies

No	Study^a^ [reference no.]	Year of publication	Study design	Country	Targeted population
1	Soe et al. [55]	2017	Cross-sectional survey	Myanmar	Internal migrants
2	Phyo Than et al. [54]	2017	Cross-sectional survey	Myanmar	Migrant workers
3	Ly et al. [53]	2017	Cross-sectional survey using RDS^b^	Cambodia	Mobile and migrant population
4	Kounnavong et al. [52]	2017	Review	Lao	In–out migrations and military personnel
5	Crawshaw et al. [51]	2017	Cluster randomised trial	Myanmar	Migrant rubber tappers
6	Zhang et al. [50]	2016	Case study	China	Fever cases in the border areas
7	Vezenegho et al. [49]	2016	Survey	French Guiana	Forest workers
8	Nyunt et al. [48]	2016	Mixed method (qualitative and quantitative)	Myanmar	Local health volunteers for migrants
9	Krisher et al. [47]	2016	Case study	South America	Cross-border migrants
10	Douine et al. [46]	2016	Prospective, multicentre	French Guiana	Illegal gold miners
11	de Santi et al. [45]	2016	Cross-sectional survey	French Guiana	Illegal gold miners
12	Charchuk et al. [44]	2016	Cross-sectional survey	Congo	Internally displaced persons
13	Canavati et al. [43]	2016	Mixed method (qualitative and quantitative)	Cambodia	Seasonal workers
14	Castellanos et al. [42]	2016	Retrospective chart review	Columbia	Illegal gold miners
15	Schicker et al. [41]	2015	Cross-sectional survey (venue based survey)	Ethiopia	Migrant workers
16	Peeters et al. [40]	2015	Cross-sectional survey	Cambodia	Migrants
17	Nyunt et al. [39]	2015	Cross-sectional survey	Myanmar	Mobile population
18	MOH, Malaysia et al. [38]	2015	Case study	Malaysia (Sabah)	Migrants
19	Hlaing et al. [37]	2015	Cross-sectional survey	Myanmar	Internal migrants
20	Wai et al. [36]	2014	Cross-sectional survey	Myanmar	Migrant workers
21	Nyunt et al. [35]	2014	Cross-sectional survey	Myanmar	Migrant workers
22	Gueye et al. [34]	2014	Case study with mixed method	Namibia	Population in the border areas
23	Obol et al. [33]	2013	Cross-sectional survey	Uganda	Internally displaced persons
24	Kirkby et al. [32]	2013	Cross-sectional survey	Sri Lanka	People in a post-conflict setting
25	Qayum et al. [31]	2012	Cross-sectional survey	Pakistan	Internally displaced persons
26	Hiwat et al. [30]	2012	Case study	Suriname	Post-conflict district
27	Burns et al. [29]	2012	Randomized trial	Sierra Leone	Refugees
28	Abeyasinghe et al. [28]	2012	Case study	Sri Lanka	People in a conflict setting
29	Wangroongsarb et al. [27]	2011	Cross-sectional survey using RDS^b^	Thailand	Migrant workers
30	Mullany et al. [26]	2010	Pre-post comparison^c^	Myanmar	Mon state
31	Lee et al. [25]	2009	Evaluation report	Myanmar	Internally displaced persons
32	Kolaczinski et al. [24]	2006	Cross-sectional survey	Uganda	Internally displaced persons
33	Carrara et al. [23]	2006	Cross-sectional survey (before, during and after interventions)	Thailand	IDP
34	Guyant et al. [22]	2015	Review	Cambodia	Mobile and migrant population
35	IOM et al. [20]	2012	Review	Myanmar	Internal MMPs
36	Zhou et al. [15]	2016	Surveillance	China	Internally displaced persons

^a^ First author of the study; ^b^ RDS: respondent-driven sampling (i.e. a sampling method based on snowball approach); ^c^ Seem as a before-after design

**Fig. 3:**
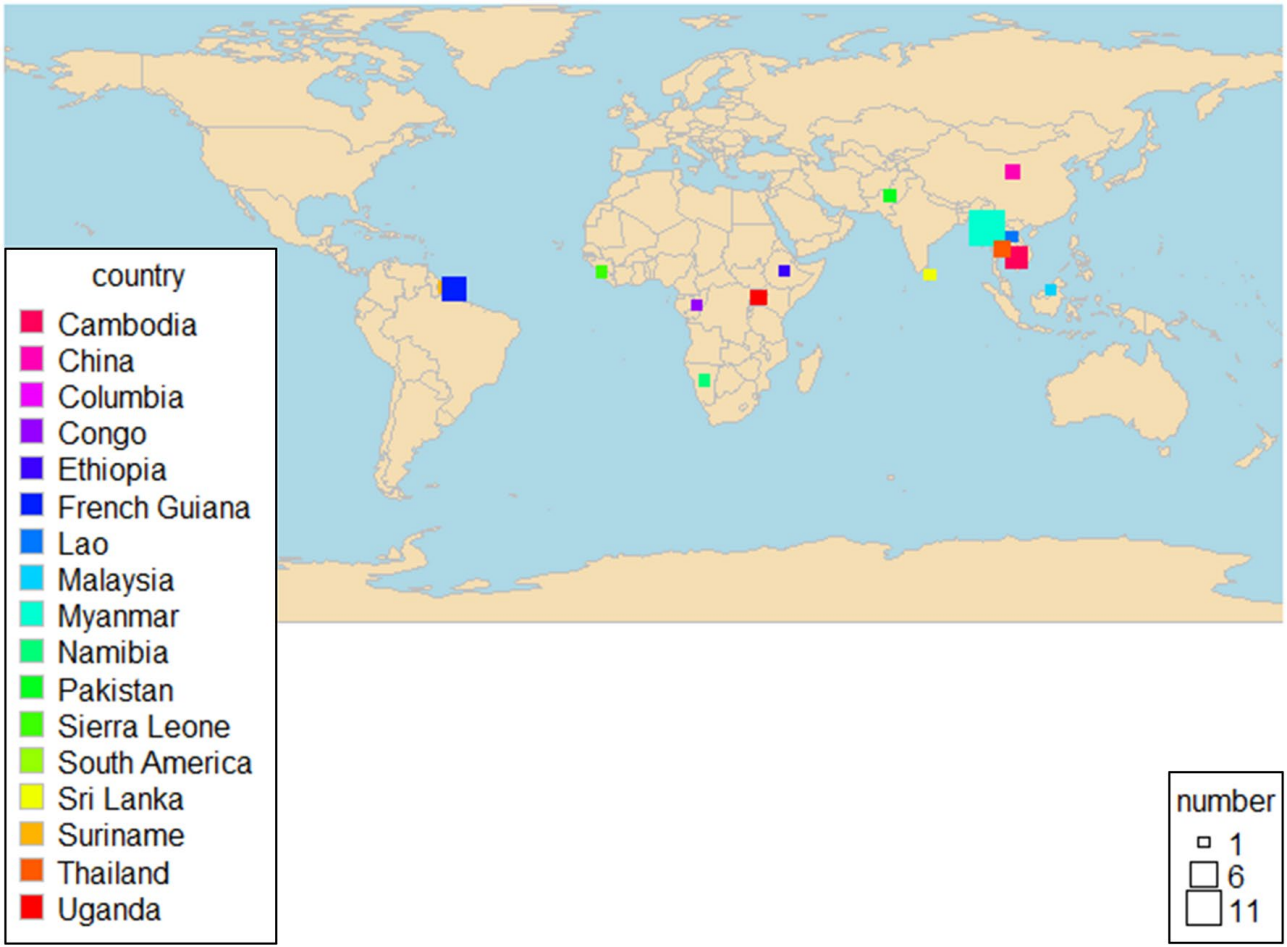
Distribution of study countries

Figure 3 shows the geographic distribution of the studies included. The key characteristics of the included studies are provided in Table 1. Eleven studies (30.5%) were from Myanmar [20, 25, 26, 35–37, 39, 48, 51, 54, 55], four studies (11.1%) from Cambodia [22, 40, 43, 52], three studies from French Guiana [45, 46, 48] and two studies each from China [15, 50], Thailand [23, 27], Sri Lanka [28, 32] and Uganda [24, 33]. The remaining ten single studies were done in Columbia [43], Congo [44], Ethiopia [41], Lao [52], Malaysia [38], Namibia [34], Pakistan [31], Sierra Leone [29], Suriname [30] and South America [47].

### Stakeholders involved

The list of stakeholders in the intersectoral actions for malaria control among MMPs is presented in Additional file 3. A variety of stakeholders, such as MOH/MOPH, other government ministries, bilateral cooperation initiatives, private sectors, international and local NGOs, and faith-based organizations, were identified for intersectoral actions to defeat malaria amongst MMPs. Almost all studies discussed the involvement of MOH/MOPH, except two studies from Myanmar in which international NGOs (INGO) and faith-based organizations appeared to be the key actors [25, 26]. The other ministries involved were the Ministry of Mines and Energy in Columbia [42] and the Ministry of Education in French Guiana [46]. Bilateral cooperation activities such as the Trans-Kunene Malaria Initiative (TKMI) between the ministries of Namibia and Angola and ‘SOSEK MALINDO’ between the ministries of Malaysia and Indonesia were also identified. However, there is dearth of information on how these stakeholders shared roles and responsibilities for implementation, the channels of communication between-and-within the partners and with the MOH/MOPH. However, all but one study [15] provided clear information on the stakeholders, the type of services provision and duration of their stay in different places.

The interventions that the stakeholders involved/supported are presented in Additional file 4. Of these 36 studies included, the most frequently assessed intervention was the coverage and utilization of insecticide-treated nets (ITNs)/long-lasting insecticide-treated nets (LLINs) as personal protective measures (PPM) (40.5%), followed by the intervention of early diagnosis and treatment of malaria (33.3%), the surveillance and response activities (13.9%) and the behaviour change communication (BCC) (8.3%).

### Lessons learned

Table 2 presents the summary of lessons learned on the intersectoral involvement for malaria control/elimination amongst MMPs. Almost all studies described the success factors for the intervention activities (e.g. ITNs), but there is limited description on which a particular agency/sector was involved and how they collaborated with each other in malaria control/elimination activities. Only three studies explicitly provided details on factors contributing towards the success of intersectoral involvement at the community level for MMPs. These were noted as: “s*trengthened partnership and established the collaboration, coordination and cooperation channels among stakeholders”* [50, p. 8], “*prompt establishment of health care clinics, resource mobilization by international agencies and NGOs in response to the disaster*” [15, p. 7] and “*receipt of a steady source of detailed, accurate, government and NGO*-*sponsored information”* [53, p. 7]. Similarly there was limited discussion on the challenges encountered. Only 2 studies explicitly described “*The* n*eed to improve mechanisms of communication among multiple partners”* [36, p. 9] and “*the assurance of long*-*term, sustainable funding”* [28, p. 11].

**Table 2:** Description of lessons learned from the intersectoral involvement for malaria control targeted to the mobile and migrant populations

Study, year	Country	Lessons learned (success)
Zhang, 2016 [50]	China	Strengthened the partnership and established the collaboration, coordination and cooperation channels among stakeholders. Health Poverty Action (HPA) is an example model
Zhou, 2016 [15]	China	Prompt establishment of health care clinics, resource mobilization by international agencies and NGOs in response to the disaster
Ly, 2017 [53]	Cambodia	Received a steady source of detailed, accurate, government and NGO-sponsored information
Zhou, 2016; [15] Carrara, 2006 [43]	China; Thailand	Significantly reduced incidence with effective management
Obol, 2015 [33]	Uganda	In all IDP camps, health care services and ITNs distribution etc. were solely provided by the emergency relief organisations and the UN
Lee, 2008 [25]	Myanmar	Feasibility of delivering effective disease control interventions in an area of active conflict through the trained volunteers
Kirkbya, 2012 [32]	Sri Lanka	Malaria is taught during grade 6 of the school curriculum, i.e. at the beginning of secondary school education
Nyunt, 2014 [35]	Myanmar	Free distribution was found as one of the major factors causing utilization of ITNs in migrant workers
Canavati, 2016 [43]	Cambodia	Targeted community was satisfied with the mobile malaria workers’ services
Lessons learned (challenges)		
Wai, 2014 [36]	Myanmar	Need to improve mechanisms of communication among multiple partners
Wai, 2014 [36]	Myanmar	Need collaborative work between health department and administrators to inform and motivate the regular use of LLINs
Abeyasinghe, 2012 [28]	Sri Lanka	The assurance of long-term, sustainable funding
Ly, 2017 [53]; Wai, 2014 [36]; Wangroongsarb, 2011 [27]; Peeters, 2015 [40]	Cambodia; Myanmar; Thailand	Limited the effectiveness of health education message/IEC due to limited literary or language barrier in multilingual ethnic groups
Ly, 2017 [53]	Cambodia	~ 10% of participants treated for malaria did not have a confirmed diagnosis
Ly, 2017 [53]; Obol, 2013 [33]; Charchuk, 2016 [44]	Cambodia; Uganda;	Low net utilization rates
Zhou, 2016 [15]	China	Interventions exclusively to IDP camps, excluding local surrounding villages
Gueye, 2014 [34]	Namibia	Not appropriate timing of the spray season; Late payment of temporary spray men may have resulted in decreased morale and lower quality of IRS
Zhou, 2016 [15]; Wai, 2014 [36]; Wangroongsarb, 2011 [27]	China; Myanmar; Thailand	Lack of convenient access to health care facilities/limited access to formal health facility/health message; Transportation constraints to access health care facility
Wai, 2014 [36]	Myanmar	A gap in willingness to buy ITNs/LLINs and affordability
Canavati, 2016 [43]		Short stay of mobile malaria workers; Low utilization of mobile malaria workers
Carrara, 2006 [23]	Thailand	2-day artesunate regimen given, not a standard 3-day regimen
MOH, Malaysia, 2015 [38]	Malaysia	Undocumented migrant workers are a challenging group to access/trace for the malaria elimination intervention
Qayum, 2012 [31]	Pakistan	Limited distribution of ITNs; No worn out bed nets were replaced; some were not in a useable state
Lee, 2009 [25]	Myanmar	Exceeded the capacity to train volunteers or to monitor and evaluate their work; Inadequate training of volunteers and a lack of strong guidelines for recruiting villagers
Lee, 2009 [25]	Myanmar	Community health workers reluctance to delegate additional responsibilities to the volunteers
Lee, 2009 [25]	Myanmar	Recruitment, training and supervision of volunteers became more time consuming for clinic staff
Lee, 2009 [25]	Myanmar	Over-treatment of test-result negative patients by volunteers
Nyunt, 2014 [35]	Myanmar	Unpleasant insecticide smell of the nets

*IDP* internally displaced people, *IRS* indoor residual spraying, *ITN* insecticide treated bed net/material, *LLIN* long lasting insecticide treated bed net/material, *NGO* non-governmental organization

### The outcome of interventions

**Fig. 4:**
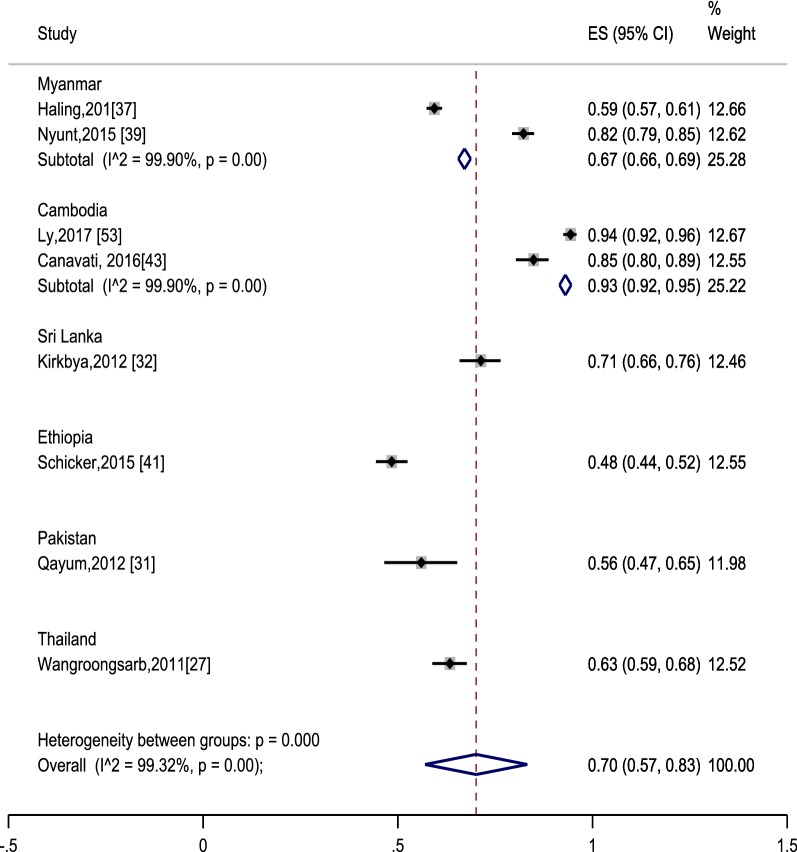
Proportion of the mobile and migrant populations who correctly know malaria as a mosquito-borne disease. Effect size (ES) indicates proportion. Each included study is represented by squares at the estimated point of effect. The horizontal lines through the square illustrate the length of the confidence interval (CI). The longer the lines, the wider the CI, the less reliable the study results. A subtotal or the overall combined result is represented by a diamond with its centre indicating the pooled point estimate, while its width representing the CI for the pooled data. The wider the width of the diamond, the less reliable the pooled results

A subset of eight studies from six countries was identified, that provided details on the proportion of MMPs with the correct knowledge about malaria as a mosquito borne disease [27, 31, 32, 37, 39, 41, 43, 53]. Overall, a pooled estimate was 70% (95% CI 57–83%), indicating a high proportion of the targeted MMPs had correct knowledge about malaria transmission (Fig. 4). There was a substantial variation within study heterogeneity, and the estimates varied from a low level 48% (95% CI 44–52%) in Ethiopia [41] to sufficient level of knowledge in Cambodia (93%, 95% CI 92–95%) [53]. Gaps were obvious even within the same country. For instance, 58% of MMPs located in the Myanmar Artemisinin Resistance Containment (MARC) zone in Bago region alone [37] and 82% of MMPs located in the MARC zone in Kayin State, Mon State, Bago region and Tanintharyi region of Myanmar [39] had correct knowledge about malaria transmission. This implied that there might be variations in modes of delivery of health education (HE) messages.

**Fig. 5:**
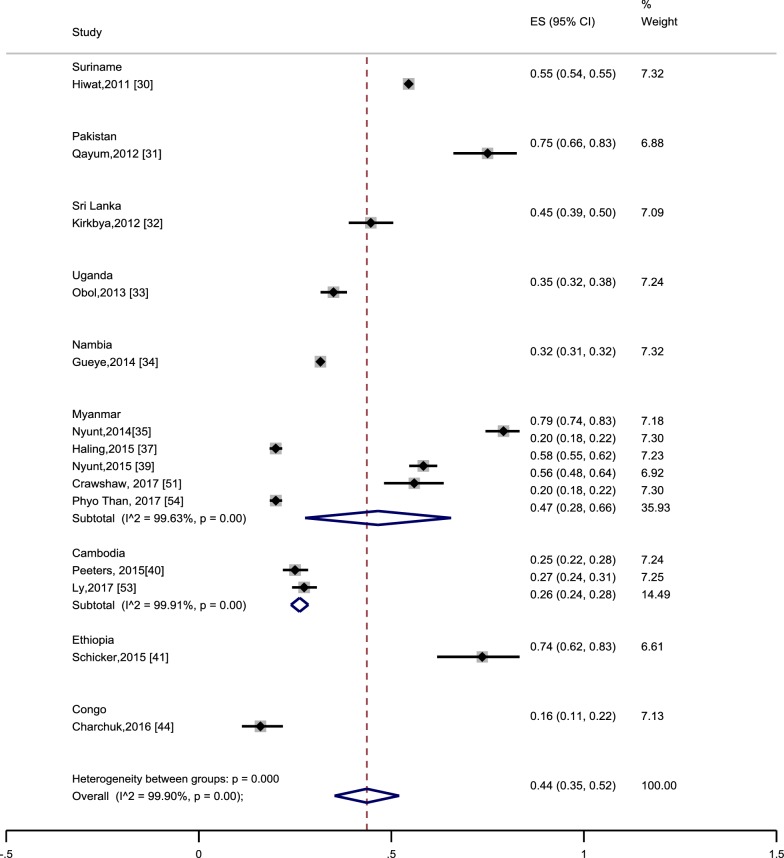
Proportion of nets ownership among the mobile and migrant populations. Effect size (ES) indicates proportion. Each included study is represented by squares at the estimated point of effect. The horizontal lines through the square illustrate the length of the confidence interval (CI). The longer the lines, the wider the CI, the less reliable the study results. A subtotal or the overall combined result is represented by a diamond with its centre indicating the pooled point estimate while its width representing the CI for the pooled data. The wider the width of the diamond, the less reliable the pooled results

Estimates of net ownership (including insecticide-treated clothing, ITC) amongst MMPs were available across fourteen studies from nine countries. Overall, a pooled estimate was 44% (95% CI 35–52%), indicating less than half of the targeted MMPs used ITNs. A subgroup of five studies conducted in Myanmar [35, 37, 39, 51, 54] also showed similar results (47%, 95% CI 28–66%) (Fig. 5). There was substantial heterogeneity (*I*^2^ 99.9%), indicating between-country and within-country variation. For instance, net utilization rate was relatively higher in studies from Pakistan (75%) [31] and Ethiopia (74%) [41], but was observed to have lower estimates in a study from Congo (16%) [44]. Qualitative studies reinforced that community acceptance of ITNs was a major factor in utilisation and vice versa. An example from Myanmar was *“I don’t know that is ITN. I don’t like it because it is too rough in texture with big pits. It looks like the nets used for animals such as buffalos and cows in my native town. Some of villagers use it to catch up fish”* [35 p. 5].

**Fig. 6:**
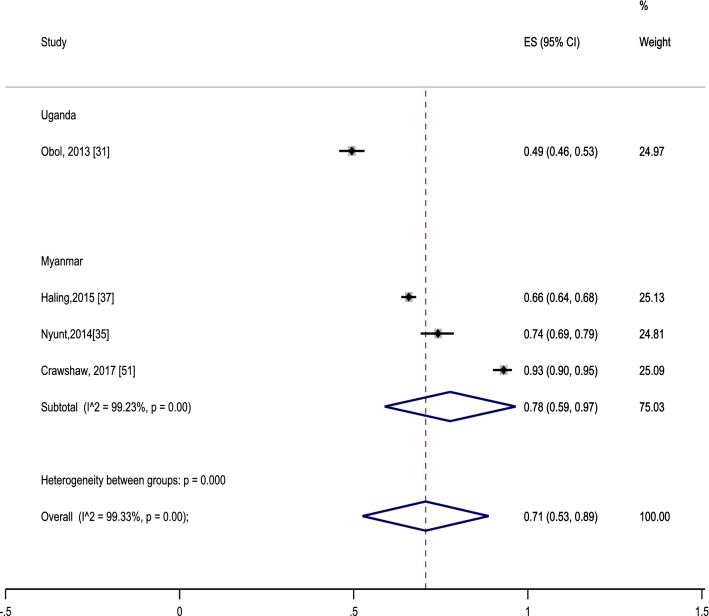
Proportion of participants with willingness to pay for insecticide treated materials/bed nets. Effect size (ES) indicates proportion. Each included study is represented by squares at the estimated point of effect. The horizontal lines through the square illustrate the length of the confidence interval (CI). The longer the lines, the wider the CI, the less reliable the study results. A subtotal or the overall combined result is represented by a diamond with its centre indicating the pooled point estimate, while its width representing the CI for the pooled data. The wider the width of the diamond, the less reliable the pooled results

Interestingly, the paradoxical phenomenon of a high proportion of MMPs with knowledge about malaria transmission, but with a low proportion of net utilization was found in a study from Cambodia [53], and vice versa in a study from Ethiopia [41]. This implied that there was a gap between knowledge acquisition and the actual practice among these MMPs. Overall, a pooled analysis of four studies [31, 35, 37, 51] showed that a high proportion of participants were willing to buy ITNs/LLINs/ITCs (71%, 95% CI 53–89%) (Fig. 6). Variation in the willingness to purchase as supported by substantial heterogeneity (99.3%) may be linked to the level of understanding of and belief in the benefits of using ITNs [33]. Interestingly, one study in Myanmar reported the gap between willingness to buy ITNs/LLINs and affordability (88.5% vs. 60.2%) [36].

**Fig. 7:**
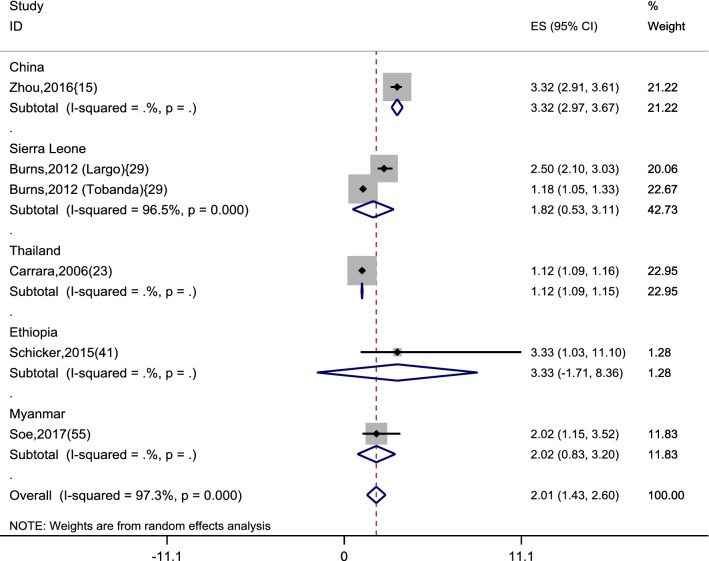
Cases reduction related to the interventions compared to no-interventions for the mobile and migrant populations. Effect size (ES) indicates odds ratio and its 95% confidence interval (CI). Each included study is represented by squares at the estimated point of effect. The horizontal lines through the square illustrate the length of the CI. The longer the lines, the wider the CI, the less reliable the study results. A subtotal or the overall combined result is represented by a diamond with its centre indicating the pooled point estimate while its width representing the CI for the pooled data. The wider the width of the diamond, the less reliable the pooled results

Among the studies that measured an outcome of malaria case reduction, five studies (with six datasets) provided data with comparable reporting methods [15, 23, 29, 41, 55] with either a comparison before and after interventions or between intervention and no-intervention. An intervention for utilization of ITNs/LLINs was two times more likely to reduce malaria incidence amongst the targeted MMPs (summary OR 2.01, 95% CI 1.43–2.6) (Fig. 7). Amongst MMPs in China-Myanmar border areas, those who reported the habit of (always) sleeping under a bed net at night were likely to have a threefold reduction in malaria incidence compared to those who did not reported this behaviour (OR 3.2, 95% CI 2.9–3.7) [15]. Only one study on the Myanmar-Thailand borders provided data on outcome of early detection and treatment. It showed a 12% increase in malaria cases in the non-intervention groups compared to those MMPs under intervention (OR 1.12, 95% CI 1.09–1.16) [23].

The current findings showed that if BCC is integrated within an intervention, rather than stand-alone, people would initiate and sustain the desired behaviours (e.g. sleeping under LLINs). For instance, the Nyunt study in Myanmar used an integrated BCC approach of HE supporting bed net distribution [39] and the outcome was a high proportion of MMPs with adequate knowledge, whereas the Hlaing study in the same country was implemented using stand-alone HE approach [37].

There were several studies on the surveillance and response approach, but they combined data on MMPs and non-MMPs or did not clearly identify intersectoral actions there. Although they were not included in the current review, a common finding in these studies was the high levels of asymptomatic malaria. The detection of asymptomatic malaria was through active case detection (ACD) and reactive case detection (RCD) activities among MMPs. One of the included studies was conducted in the illegal gold mining population of French Guiana. It showed that RDTs and microscopy (used for surveillance) did not identify all the people who had malaria parasites [46]. Compared to PCR, the RDT sensitivity was very low (16%, 95% CI 9.9–27.7%) as was microscopy (18%, 95% CI 11.6–27.1%). However, specificity was very high with RDT (99.1%, 95% CI 97.3–99.7%) or with microscopy (100%, 95% CI 98.8–100%). This would mean that 84% and 82% of humans carrying malaria parasites would have been missed by using only microscopy or only RDT, respectively. Several stakeholders, such as MOH, regional health agencies, the French Army Health Department, the Ministry of Foreign Affairs, the Home Affairs Ministry, the Overseas Territories Ministry, PAHO/WHO and the Global Fund, supported the interventions targeted to these high risk population in French Guiana [49]. There were very limited descriptions of the roles and responsibilities of communication channels between the stakeholders and with public sectors of the host country that can helped others adopt or adapt these approaches.

## Discussion

The present review summarizes thirty-six studies across seventeen countries. This is the first systematic review which assessed intersectoral collaboration for malaria control targeted to MMPs in pre-elimination or elimination phases. “*Intersectoral action is a strategy used to deal with complex policy problems that cannot be solved by a single country, region, government, department, or sector. Intersectoral action has been brought to bear on specific determinants of health, diseases, populations (e.g. indigenous peoples, children), geographic communities, health behaviours, and risk factors”* [56, p. 7].

### The major observations in this review are

- Malaria is a health problem amongst MMPs, including mining communities, who had limited access to formal healthcare facilities and low utilization of PPMs such as ITNs;
- Multiple stakeholders including public sectors, local and international agencies, NGOs, private sectors, employers of concern had been supporting the various interventions for malaria control/elimination targeted to these high risk populations;
- Although limited details were provided in the studies, the intermediate outcomes showed some evidence that the intersectoral collaborations contributed to the improvement in knowledge about malaria. This also initiated and promoted bed net utilization; increased access to diagnosis and treatment interventions and contributed towards a reduction in malaria incidence.

### The need for more detailed description of partnerships

Intersectoral collaboration to address health problems was described 50 years ago in the Alma Ata Declaration of 1978 [56]. The current review identified several agencies who played various roles such as suppliers of materials, provider of services or research collaborators through intersectoral approaches who targeted the MMPs. However, there was inadequate description and limited robust analysis of the contribution of these intersectoral actions made towards achieving the targeted malaria control outcome. This was because these studies were designed to address their specific objectives, rather than to undertake a robust assessment of the intersectoral collaboration. Future studies are needed which are designed to assess the role of intersectoral interventions targeted to MMPs.

Although there was paucity of data, evidence was found that interventions targeted towards malaria in IDP camps/amongst MMPs could reduce malaria incidence/prevalence significantly in comparison to the surrounding villages or those villages without an intervention. Moreover, studies included in this review had highlighted the important role of intersectoral actions. An example from a study in Uganda was… “*In all IDP camps, health care services and ITN distribution* etc*. were solely provided by the emergency relief organisations and the UN was* [33 p. 963]. In fact, the intersectoral collaboration is required *“because of the wide range of interests involved, additional effort and negotiation to reach a shared understanding of goals, approaches, respective roles, and accountability for outcomes”* [57].

It seemed that conditions in illegal mining camps in French Guiana showed less success against their desired outcomes that in other MMPs settings. A reason for this might be related to multiple factors including the complexities involved in accessing these populations, and because investing in “illegal” miners’ health was not sanctioned or funded [46].

### Better segmentation of behaviour change communication is of immense value

MMPs were often targeted by interventions as a homogenous group, but in reality they have varying health beliefs, patterns of health behaviour and utilization of health services [18]. The current analysis confirmed this assertion by revealing the geographical variations in the level of knowledge about malaria transmission or net utilization. This difference was possibly related to type of interventions for MMPs. For instance, BCC is a term often used to describe any communication strategy with individuals or communities to promote positive behaviours appropriate to their settings.

The proportion of people equipped with knowledge about malaria transmission through the bite of (infective female) mosquitoes was higher, but the net utilization rates (ITNs/LLINs) were still at inadequate levels for personal protection. This implied that there was a gap between the knowledge and the actual practice amongst these populations. These discrepancies suggested a range of areas for investigation and improved interventions. For example, it needs well-designed BCC coupled with improved accessibility to, and affordability of, the means of protection in order to support people to convert their knowledge and supportive attitudes into malaria control practice. Moreover, other aspects of a supportive environment such as community and health services support, innovative methods of newer or modified means of protection and treatment that is acceptable, affordable and convenient for the population [36] must be included in an intervention.

In addition, factors linked to the social determinants of health such as income and education are often the strongest predictors of bed net use [51, 58]. An analysis of these factors was beyond the scope of this review but would be useful to undertake in future studies.

### Broadening access to all beyond the MMPs

Interventions identified for the current review seemed to be designed by the partners/agencies/donors to specifically serve the IDP camps/MMPS, and excluded neighbouring villages. In the elimination phase, all instances of detected parasitaemia (including gametocytaemia only) are considered as ‘malaria case’ as they might lead to onward transmission, regardless of the presence or absence of clinical symptoms [59]. Hence, it is crucial to expand malaria intervention strategies in IDP camps to local surrounding villages in the border area [22]. The malaria control strategy in the critical period of pre/elimination phase in the areas of MMPs should be an “*all inclusive*” approach, by expanding services to those non-MMPs who share the same tyrannies of poor access to health services and programmes. However, there might be limitations in the ‘agency’ mission, funding and approvals that will not support this broadening of the target populations. Finding ways to scale up successful interventions utilized for MMPs to broader catchments may need different collaborations and should be studied.

### Sustainability issues

An important issue related to the current findings was the sustainability of the agency/donor-dependent interventions. For instance, there was a gap between the willingness and ability of the populations to pay for ITNs [37], which would become the case should donor funding cease. This is of great concern as most of the MMPs within the border areas are poor and they have limited employment opportunities [58]. More detailed consideration of sustainability of malaria control interventions among various sub-populations of MMPs is required to achieve the targets of elimination and sustaining them in those populations and susceptible contiguous regions.

### Study limitations

The findings of the intersectoral activities and the outcomes were exclusively based on research studies conducted in the IDP camps or areas where MMPs reside. It appears that the studies included in this review were not designed to study the outputs or outcomes from the processes for intersectoral approaches even when these approaches were the major platform for delivery of the intervention (e.g. ITN distribution or BCC activities). It is likely that countries have developed strategies for malaria control activities with MMPs through intersectoral actions that have not been published and are, therefore, not included in this review. Moreover, the reported findings could be geographically biased due to an unequal number of included studies and limited to generalizability towards MMPs/IDPs across countries.

Regarding the methodology, there was substantial heterogeneity among studies (*I*^2^: 97.3%). The fact that *I*^2^ value remained high in the meta-analysis implied that there might be factors inherent in the included studies; the individual characteristics of MMPs, migration patterns, level of malaria endemicity in their localities, the presence of co-infections/co-morbid conditions, and coverage of effective malaria interventions. Due to inadequate data, stratified analyses based on all these influencing factors were not possible. Future studies should consider these factors in their design.

### Public health implications

Universal health coverage must be the goal for all people at risk of malaria including MMPs. Control of malaria and effective treatment was problematic since it was difficult for routine health sector activities, especially public sector, to locate, diagnose and treat infected people in these populations. Malaria programmes can adapt the methods of a wide-reaching “pre-surveillance assessment” process that has been done in the HIV programmes, as describe elsewhere [60]. Access to early diagnosis and effective treatment by promoting ACD and RCD and/or provision of innovative clinical treatment models such as mobile clinics are crucial for these populations. Moreover, the use of PPM and available healthcare services could be maximized through improved knowledge and supportive attitudes towards malaria control supported by effective BCC that were linked to improve provision of required “tools” for PPM.

It is important to segment health communications to address the specific language, cultural, gender specific, contextual and literacy needs in the MMPs. Well-developed and evidence-based IEC and BCC that are based on the needs, characteristics and culture of the MMPs including migrant workers are needed to increase knowledge of symptoms, prevention and control measures. In addition, sources and need for early diagnosis-based treatment and care and the risks associated with delays in treatment also need to be addressed. Community-based interventions and services through a network of village health workers and community volunteers to strengthen malaria prevention and control measures might be particularly useful for the MMPs who have limited access to health services [1, 27, 57].

## Conclusions

The findings suggest that interventions supported by multiple stakeholders have a significant impact on reduction of malaria transmission in the targeted MMPs. It is important to realize that intersectoral action is a key strategy for various interventions targeted to those populations not usually reached by routine health services. A well-coordinated strengthened partnership of multiple stakeholders including employers of the targeted MMPs, public health sectors, other related ministries, private medical sectors and implementing NGOs is urgently needed to enhance the outcome of malaria control and elimination efforts targeting these often neglected and underserved populations. Well-designed studies from different countries to robustly assess the role of intersectoral interventions targeting the MMPs and the impact on the reduction of transmission are recommended.

## Additional files

**Additional file 1.** PRISMA checklist.

**Additional file 2.** Excluded studies and the reasons for exclusion.

**Additional file 3.** List of the stakeholders involved for malaria control among mobile and migrant populations.

**Additional file 4.** Intersectoral involvement in the malaria intervention activities targeted to the mobile and migrant populations.

## Abbreviations

ACD: active case detection
BCC: behaviour change communication
HE: health education
HPM: human population movement
HPA: Package of Health Poverty Action
IDP: internally displaced persons
IEC: information, education and communications
IOM: International organization for migration
ITC: insecticide treated clothing
ITN: insecticide treated nets
LLINs: long lasting insecticide treated nets
MARC: Myanmar Artemisinin Resistance Containment
MMP: mobile and migrant population
MOH: Ministry of Health
MOPH: Ministry of Public Health
NGO: non-governmental organizations
PCC: population, context, and contents
PPM: personal protective measures
PRISMA: Preferred Reporting Items for Systematic Reviews and Meta-Analyses
PSI: Population Services International
RBM: Roll Back Malaria
RCD: reactive case detection
RDT: rapid diagnostic test
TKMI: Trans-Kunene Malaria Initiative
UNDP: United Nations Development Programme
UNHCR: United Nations High Commission for Refugees
UNICEF: United Nations Children’s Fund
WHO: World Health Organization
