# Supplementary material for: Inter-sectoral approaches for the prevention and control of malaria among the mobile and migrant populations: a scoping review
Source: Malar J. 2018 Nov 16;17:430. doi: 10.1186/s12936-018-2562-4 (PMC6240188; doi:10.1186/s12936-018-2562-4)
Supplement: Supplementary file 2 — Additional file 2. Excluded studies and the reasons for exclusion. [file 12936_2018_2562_MOESM2_ESM.doc]

Additional file 2. Excluded studies and the reasons for exclusion

| First author, year of publication | Main reasons for exclusion | Full citations |
| --- | --- | --- |
| Chaveepojnkamjorn, 2004 | Not indicated intersectoral actions | Chaveepojnkamjorn W, Pichainarong N. Malaria infection among the migrant population along the Thai-Myanmar border area. Southeast Asian J Trop Med Public Health. 2004; 35:48-52. |
| Moore, 2008 | No specific data for intersectoral involvement | Moore SJ, Min X, Hill N, et al. Border malaria in China: knowledge and use of personal protection by minority populations and implications for malaria control: a questionnaire-based survey. BMC Public Health. 2008; 8:344. |
| Khamsiriwatchara,2011 | Not on malaria | Khamsiriwatchara A, Wangroongsarb P, Thwing J, et al. Respondent-driven sampling on the Thailand-Cambodia border. I. Can malaria cases be contained in mobile migrant workers? Malar J. 2011;10:120 |
| Breeveld, 2012 | Not specific to MMPs | Breeveld FJ, Vreden SG, Grobusch MP. History of malaria research and its contribution to the malaria control success in Suriname: a review. Malar J. 2012;11:95. |
| Cao,2014 | 137 surveillance system, Multi-sectors, but not with MMPs | Cao J, Sturrock HJW, Cotter C, et al. Communicating and monitoring surveillance and response activities for malaria elimination: China’s ‘‘1-3-7’’ strategy. PLoS Med .2014. 11(5): e1001642. |
| Pindolia, 2014 | Not indicated intersectoral actions | Pindolia DK, Garcia AJ, Huang Z, et al. Quantifying cross-border movements and migrations for guiding the strategic planning of malaria control and elimination. Malar J. 2014; 13:169. |
| Tizzoni, 2014 | Not an empirical study, but a modelling study | Tizzoni M, Bajardi P, Decuyper A, et al. On the use of human mobility proxies for modeling epidemics. PLoS Comput Biol. 2014;10:e1003716 |
| Nyunt,2015 | Molecular surveillance, not indicated intersectoral actions/ MMPs | Nyunt MH, Shein T, Zaw NN, et al. Molecular evidence of drug resistance in asymptomatic malaria infections, Myanmar, 2015. Emerg Infect Dis. 2017;23:517-520. |
| Rosas-Aguirre, 2015 | Not specific to MMPs | Rosas-Aguirre A, Speybroeck N, Llanos-Cuentas A, et al. Hotspots of malaria transmission in the Peruvian Amazon: rapid assessment through a parasitological and serological survey. PLoS One. 2015;10:e0137458. |
| Zhou,2015 | “1-3-7”, not clear about the intersectoral involvement or MMPs | Zhou SS, Zhang SS, Zhang L, et al. China's 1-3-7 surveillance and response strategy for malaria elimination: Is case reporting, investigation and foci response happening according to plan? Infect Dis Poverty. 2015;4:55. |
| Chihanga, 2016 | “1-3-7”, not clear about the intersectoral involvement | Chihanga S, Haque U, Chanda E, et al. Malaria elimination in Botswana, 2012-2014: achievements and challenges. Parasit Vectors. 2016;9:99. |
| de Santi, 2016 | No specific data for intersectoral involvement | de Santi V P, Girod R, Mura M, et al. Epidemiological and entomological studies of a malaria outbreak among French armed forces deployed at illegal gold mining sites reveal new aspects of the disease's transmission in French Guiana. Malar J. 2016;15:35 |
| Feng,2016 | “1-3-7”, not clear about the multisector involvement or MMPs | Feng J, Liu J, Feng X, et al. Towards malaria elimination: monitoring and evaluation of the "1-3-7" approach at the China-Myanmar border. Am J Trop Med Hyg. 2016;95:806-10. |
| Gueye, 2016 | Some overlapped data/information with an included study | Gueye C S, Newby G, Tulloch J, et al. The central role of national programme management for the achievement of malaria elimination: a cross case-study analysis of nine malaria programmes. Malar J. 2016;15:488 |
| Lu, 2016 | “1-3-7”, not clear about the multisector involvement or MMPs | Lu G, Liu Y, Beiersmann C, et al. Challenges in and lessons learned during the implementation of the 1-3-7 malaria surveillance and response strategy in China: a qualitative study. Infect Dis Poverty. 2016;5:94. |
| PAHO, 2016 | Action plan for IDPs/MMPs | PAHO. Plan of action for malaria elimination 2016-2020. USA, Washington DC. 2016. |
| Recht,2017 | No specific information about MMPs | Recht J, Siqueira AM, Monteiro WM, et al. Malaria in Brazil, Colombia, Peru and Venezuela: current challenges in malaria control and elimination. Malaria J. 2017;16:273. |

MMP: Mobile and migrant population
