# Supplementary material for: Inter-sectoral approaches for the prevention and control of malaria among the mobile and migrant populations: a scoping review
Source: Malar J. 2018 Nov 16;17:430. doi: 10.1186/s12936-018-2562-4 (PMC6240188; doi:10.1186/s12936-018-2562-4)
Supplement: Supplementary file 3 — Additional file 3. List of the stakeholders involved for malaria control among mobile and migrant populations. [file 12936_2018_2562_MOESM3_ESM.doc]

Additional file 3 List of the stakeholders involved for malaria control among mobile and migrant populations

| Type of sectors | Name |
| --- | --- |
| Government Sectors | Ministry of Health (MOH) |
|  | Ministry of Public Health (MOPH) |
|  | Ministry of Labor |
|  | Ministry of Education |
|  | Ministry of Mines & Energy |
| UN Agencies |  |
|  | United Nations High Commissioner for Refugees (UNHCR) |
|  | United Nations Children's Fund (UNICEF) |
|  | The United States Agency for International Development (USAID) |
|  | International Organization for Migration (IOM) |
|  | ECHO: Humanitarian Aid department of the European Commission (ECHO) |
|  | World Health Organization (WHO) |
|  | Pan American Health Organization (PAHO) |
| International Organizations |  |
|  | Asia Pacific Malaria Elimination Network (APMEN) |
|  | The Asian Collaborative Training Network for Malaria |
|  | Centres for Disease Control and Prevention (CDC) |
| Binational cooperation |  |
|  | Trans-Kunene Malaria Initiative (TKMI) |
|  | SOSEK MALINDO (Malaysia-Indonesia ministries) |
|  |  |
| International NGOs |  |
|  | Malaria consortium |
|  | Population Services International (PSI) |
|  | Department for International Development (DFID) |
|  | World Vision Myanmar (WVM) |
|  | University Research Co. (URC) |
|  | Family Helath Information (FHI360) |
|  | Rockefeller foundation |
|  | Medecins Sans Frontieres (MSF) |
|  | Health Poverty Action (HPA) |
| Private sectors |  |
|  | Private farms |
|  | Private labs/ clinic/GP |
| Volunteers |  |
|  | Volunteer health worker (VHW) |
|  | Volunteer malaria worker (VMW) |
|  | Community |
| Local NGOs |  |
|  | Myanmar Medical Association (MMA) |
|  | Myanmar Health Assistants Association (MMHA) |
|  |  |
| Faith-related Organizations |  |
|  | Shanghai Baptist Church |
|  | Backpack Health Worker Team (BPHWT) |

Note: The lists of agencies/sectors are not exhaustive.
