# Supplementary material for: Inter-sectoral approaches for the prevention and control of malaria among the mobile and migrant populations: a scoping review
Source: Malar J. 2018 Nov 16;17:430. doi: 10.1186/s12936-018-2562-4 (PMC6240188; doi:10.1186/s12936-018-2562-4)
Supplement: Supplementary file 4 — Additional file 4. Intersectoral involvement in the malaria intervention activities targeted to the mobile and migrant populations. [file 12936_2018_2562_MOESM4_ESM.doc]

**Addition file 4. Intersectoral involvement in the malaria intervention activities targeted to the mobile and migrant populations**

| First author, year of publication  [Ref. No] | Country | Intervention activities^α^ | Stakeholders | | | | | | | | Outcome | Remark |
| --- | --- | --- | --- | --- | --- | --- | --- | --- | --- | --- | --- | --- |
|  |  |  | MOH* | Other Gov^λ^ | Other^λλ^ | VW | private sector | Local NGO | INGO | UN |  |  |
| Crawshaw,  2017 [51] | Myanmar | 2 | √ |  |  |  |  |  | √ |  | Adequate level of KAP, high acceptability of ITCs | ITC;  Malaria Consortium |
| Kounnavong,  2017 [52] | Lao | 1,2,3 | √ |  | √ | √ |  |  |  |  | Challenging factors | Armed force |
| Ly,  2017 [53] | Cambodia | 5 | √ |  |  | √ | √ | √ | √ | √ | Services utilization rates | GPs, WHO |
| Phyo Than,2017 [54] | Myanmar | 3,5 | √ |  |  | √ |  |  |  | √ | Services utilization rates | WHO |
| Soe,  2017 [55] | Myanmar | 3,5 | √ | √ |  |  | √ |  |  |  | LLINs coverage/utilization rates | Local administrators, employers |
| Canavati,2016  [43] | Cambodia | 3,5,6 | √ | √MMWs |  |  |  | √ |  | √ | Services utilization rates |  |
| Castellanos,  2016 [42] | Columbia | 4 | √ | √ |  |  |  |  |  |  |  | MO mines & energy |
| Charchuk,  2016 [44] | Congo | 3 | √ |  |  | √ |  |  | √ |  | Low net utilization rate, high malaria prevalence |  |
| de Santi,2016  [45] | French Guiana | 1 | √ | √ |  |  |  |  |  |  | malaria prevalence | Armed force |
| Douine,2016  [46] | French Guiana | 1,3 | √ | √ |  | √ |  |  |  |  | Asymptomatic case detection | MO education |
| Krisher,2016 [47] | South America | 1,4 | √ |  |  |  | √ |  | √ | √ | Health impact | Private labs; Rockefeller foundation |
| Nyunt,2016 [48] | Myanmar | 1 | √ |  |  |  |  | √ | √ |  | RDT used rate | PSI, WVM, |
| Vezenegho,  2016[49] | French Guiana | 3 | √ |  |  | √  (c) |  |  |  |  | Net coverage & utilization rate |  |
| Zhang,2016  [50] | China | 1,2,3,4 | √ | √ |  |  |  | √ | √ | √ | Effectiveness & impact assessment | GFATM;  HPA |
| Zhou,2016  [15] | China | 4 | √ |  |  |  |  | √ | √ | √ | IDP camps vs neighbouring villages | UNHCR |
| Hlaing,2015  [37] | Myanmar | 5 | √ |  |  | √ | √ | √ | √ |  | level of KAP | GPs |
| Peeters,2015  [40] | Cambodia | 1,3 | √ |  |  | √ |  |  |  |  | Net coverage & utilization rate |  |
| Guyant,2015  [20] | Cambodia | 1,3 | √ |  |  |  |  |  |  | √ | Framework |  |
| Malaysian MOH, 2015 [38] | Malaysia (Sabah) | 2,3 | √ |  | √ |  |  |  |  | √ |  | binational  with Indonesia SOSEK MALINDO, APMEN |
| Nyunt,2015  [39] | Myanmar | 3,6 | √ |  |  | √ |  |  |  |  | Net coverage & utilization rate |  |
| Schicker,2015  [41] | Ethiopia | 1 | √ | √ |  | √ |  |  | √ |  |  | MSF, other ministries |
| Gueye,2014  [34] | Namibia | 1,2,34 | √ |  | TKMI |  |  |  |  |  | ↓ cases incidence | bilateral |
| Nyunt,2014  [35] | Myanmar | 3 | √ |  |  |  |  | √ | √ |  | Net coverage & utilization rate | MARC programme |
| Wai,2014  [36] | Myanmar | 1,3,5 | √ |  |  |  |  | √ | √ |  | Level of KAP, net coverage & utilization ate | MMA, DFID, PSI, WVM |
| Kirkby,2013  [32] | Sri Lanka | 5 | √ |  |  |  | √ | √ |  |  | Level of KAP | GPs, Civil society |
| Obol,2013  [33] | Uganda | 3 | √ |  |  |  |  | √ | √ |  | Net coverage & utilization rate |  |
| Abeyasinghe,  2012 [28] | Sri Lanka | 1,2,3 | √ |  | √ |  |  |  |  | √ |  | UCSF, Global Fund |
| Burns,2012  [29] | Sierra Leone | 3 | √ |  |  |  |  |  |  | √ | Incidences comparisons$ | UNHCR, ECHO; |
| Hiwat,2012  [30] | Suriname | 2,3,4,5,6 | √ |  |  |  |  |  |  | √ | Malaria related performance indicators | GFATM |
| IOM,2012  [18] | Myanmar | NA | √ |  |  |  |  |  | √ |  | Situation analysis | MSF, PSI, WV |
| Qayum,2012  [31] | Pakistan | 3,4 | √ |  |  |  |  |  |  | √ | Level of KAP, net coverage & utilization rate | WHO, World Bank, UNICEF, UNDP |
| Wangroongsarb,2011  [27] | Thailand | 1,5 | √ | √ |  | √ |  |  |  |  |  | MO Labour |
| Mullany,2010  [26] | Myanmar | 1 |  |  | √ |  |  | √ | √ |  | Impact assessment |  |
| Lee,2009  [25] | Myanmar | 2,3,5 |  |  | √ |  | √ | √ | √ |  |  | KDHW,BPHWT |
| Carrara,2006  [23] | Thailand | 1,4 | √ | √ |  | √ |  | √ | √ |  | ↓ cases incidence | Universities |
| Kolaczinski,  2006 [24] | Uganda | 1 | √ |  |  |  |  |  |  | √ | Adherence to treatment | RDT; WHO/RBM |

^α^activities assessed by the study team; *MOH positioned NMCP/VBDC; λ:Government ministries, except MOH; λλ: Binational collaboration or international organization; ITN: Insecticide treated nets also included insecticide treated clothing (ITC) and long lasting insecticide treated nets (LLINs); ^$^ intervention site versus non-intervention site; MO: Ministry of ;

1= EDP; 2=VC, 3=PPE/ITNs/LLINs, 4=surveillance & treat; 5=IEC/HE; 6=BCC
